# Rapid uropathogen identification using surface enhanced Raman spectroscopy active filters

**DOI:** 10.1038/s41598-021-88026-9

**Published:** 2021-04-22

**Authors:** Simon D. Dryden, Salzitsa Anastasova, Giovanni Satta, Alex J. Thompson, Daniel R. Leff, Ara Darzi

**Affiliations:** 1 Department of Surgery and Cancer, Imperial College London, St Mary’s Hospital, 10Th Floor, QEQM Wing, London, W2 1NY UK; 2 Hamlyn Centre for Robotic Surgery, Imperial College London, London, SW1 2AZ UK; 3 Department of Infection, Imperial College NHS Trust, London, W6 8RF UK; 4 Department of Surgery and Cancer, Imperial College London, St Mary’s Hospital, 2nd Floor, Paterson Building, London, W2 1NY UK

**Keywords:** Urinary tract infection, Biosensors, Biosensors, Biophotonics

## Abstract

Urinary tract infection is one of the most common bacterial infections leading to increased morbidity, mortality and societal costs. Current diagnostics exacerbate this problem due to an inability to provide timely pathogen identification. Surface enhanced Raman spectroscopy (SERS) has the potential to overcome these issues by providing immediate bacterial classification. To date, achieving accurate classification has required technically complicated processes to capture pathogens, which has precluded the integration of SERS into rapid diagnostics. This work demonstrates that gold-coated membrane filters capture and aggregate bacteria, separating them from urine, while also providing Raman signal enhancement. An optimal gold coating thickness of 50 nm was demonstrated, and the diagnostic performance of the SERS-active filters was assessed using phantom urine infection samples at clinically relevant concentrations (10^5^ CFU/ml). Infected and uninfected (control) samples were identified with an accuracy of 91.1%. Amongst infected samples only, classification of three bacteria (*Escherichia coli*, *Enterococcus faecalis*, *Klebsiella pneumoniae*) was achieved at a rate of 91.6%.

## Introduction

150 million people worldwide are diagnosed with one or more urinary tract infections (UTIs) each year, making this one of the most commonly acquired infections in humans^1–3^. The ubiquity of UTIs rapidly translates into a significant health burden, with UTIs accounting for 10.5 million healthcare visits in the United States alone, of which a fifth are to emergency departments^4^. UTIs account for $6 billion in worldwide direct healthcare costs annually^2^. Furthermore, UTIs predispose patients to severe and life-threatening conditions, with the risk of pyelonephritis increased by a factor of 4.4^5^. Similarly, one tenth of sepsis cases arise from a urinary source, a condition with an 8% mortality rate^6^.

Current diagnostics for UTIs have significant limitations, which further exacerbates the burden caused by UTIs. Screening tests such as urinalysis, while rapid, are inaccurate with sensitivities and specificities of 80%^7,8^. This leads to delayed recognition in 20% of infected cases and unnecessary treatment in 20% of uninfected cases^8–11^. Furthermore, the inability of screening tests to identify the causative bacteria necessitates treatment with broad spectrum antimicrobials, known to have worse adverse effect profiles, mediated by host microbiome disruption, as well as a propensity to induce antimicrobial resistance (AMR)^12–14^. Gold standard testing through microscopy, culture and sensitivity (M,C&S), on the other hand, provides accurate microbial classification but incurs significant delays with as long as 72 h from specimen retrieval to results^7,15^.

Raman spectroscopy uses the inelastic scattering of light to provide whole organism fingerprinting and has demonstrable accuracy for bacterial sub-classification^16–18^. However, weak bacterial Raman signals have necessitated technical and time-consuming processing steps^19–22^. Additionally, the majority of published works on uropathogen identification involve Raman microscopes as opposed to Raman spectroscopy. Whilst Raman microscopy offers the advantage of fine control of a narrow spot size as low as 1 μm, therein allowing for the collection of spectra from individual cells without competing signal from the background medium^23^, their high cost and large physical footprint will not allow their use in the clinical setting. Moreover, identification of pathogens on which to focus the laser is time-consuming and requires technical expertise. In one study, 10–30% of collected spectra needed to be sorted out due to suboptimal focusing^24^. Finally, as the user is required to focus the system onto the pathogen, Raman microscopes cannot distinguish infected from uninfected samples but are rather limited to classifying pathogens in samples already known to be infected. Work performed by Yang et al. has demonstrated Raman spectroscopy can accurately identify uropathogens after a multistep process of urine separation through centrifuge and washing followed by bacteriologic capture using electrostatic forces^22^.

Surface enhanced Raman spectroscopy (SERS) provides Raman signal enhancement through the surface plasmon resonance effect afforded by the close application of metal nanostructures to the microbes^25,26^. The SERS effect is strongly distance dependent, and as such a key challenge is to ensure close and consistent application of the microbes to the SERS-active surface^25^. Membrane filtration is a microbiologic technique used to concentrate bacteria. A number of approaches have been used to combine membrane filtration with SERS for detection and identification of bacteria^27–30^. However, the fabrication procedures used are typically complex, often involving separate preparation of metallic nanoparticles for SERS that are applied to the captured bacteria after filtration. Furthermore, where SERS-active surfaces have been directly fabricated on filters^31^, the coating thicknesses have not been adequately optimized and the diagnostic potential has not been validated.

In this article, we report the application of a gold coating to polyvinylidene fluoride (PVDF) membranes using physical vapour deposition (PVD) to create SERS-active filters. This user-friendly, single-step process allows the coated membrane filters to perform three functions: capturing bacteria, separating them from the background medium (urine), and providing Raman signal enhancement. We optimized the thickness of the gold coating to achieve maximum SERS signal intensities while retaining the filtration capabilities. Then, by applying the filters in conjunction with vacuum filtration, we demonstrated the capability for detection and identification of bacteria in urine with high sensitivity and specificity using a commercially available handheld Raman spectrometer. Importantly, this was achieved without any pre-processing of the urine samples beyond vacuum filtration through the SERS-active membranes, demonstrating the potential of this approach for future use as a point-of-care diagnostic.

## Results

### SERS-active filters—optimization of gold coating thickness

**Figure 1:**
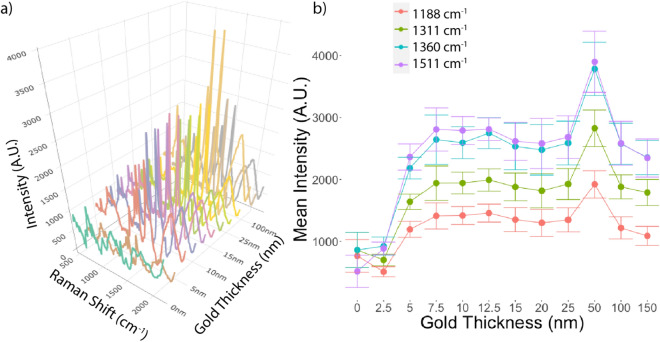
Effect of gold coating thickness on Rhodamine 6G Raman spectra. (**a**) Mean Raman Spectra of Rhodamine 6G on PVDF filters with differing gold coating thicknesses. (**b**) Mean intensity with standard deviation (error bar) of the four most prominent peaks in the Rhodamine 6G spectra (1188, 1311, 1360 and 1511 cm^−1^) for differing gold coating thicknesses. Thickness greater than 2.5 nm provided significant enhancement of all 4 prominent peaks, with the greatest enhancement seen with filters with a 50 nm gold coating.

The mean spectra of Rhodamine 6G on filters with varying gold coating thicknesses are plotted in Fig. 1a. The mean intensities of the four most prominent spectral peaks are presented in Table 1 and depicted in Fig. 1b. PVDF filters with a 50 nm gold coating applied through sputtered PVD provided the optimal signal enhancement for Rhodamine 6G, with clearly defined peaks significantly above those observed in spectra acquired from unsputtered filters and filters with different coating thicknesses (Fig. 1). Furthermore, the signal intensities were highest with the 50 nm coating for each of the four most prominent peaks in the spectra (see Fig. 1 and Table 1). As such, all further experiments were performed using 0.45 μm pore size PVDF filters with a 50 nm gold coating applied through sputtered PVD.

**Table 1:** Rhodamine 6G peak intensities observed with different gold coating thicknesses for the four most prominent spectral peaks.

Raman Peak (cm^-1^)	Peak Assignment^32^	Filter Gold Thickness (nm)										
		2.5	5	7.5	10	12.5	15	20	25	50	100	150
		Peak intensity (a.u.)										
1188	Xanthene Ring Deformation, C-H Bend, N–H bend	509	1194	1412	1418	1457	1351	1300	1348	1922	1218	1089
1311	Xanthene Ring Bending, N–H Bending@ CH_2_ Wagging	700	1641	1292	1943	1993	1880	1818	1927	2829	1881	1789
1360	Xanthene Ring Stretching, C–H Bending	862	2183	2645	2595	2750	2534	2481	2590	3788	2579	2354
1511	Xanthene Ring Stretching, C–N Stretching	515	2364	2809	2793	2812	2619	2583	2684	3903	2585	2347

#### Raman assessment of phantom urine infections

**Figure 2:**
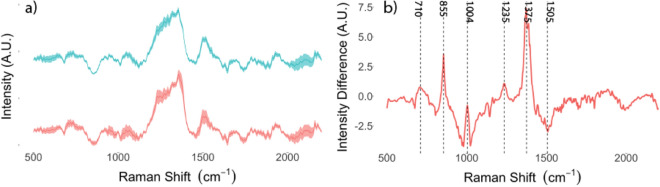
(**a**) Mean normalized Raman spectra and standard deviations (shaded areas) for infected (red) and uninfected (blue) urine samples captured on SERS-active filters. (**b**) Mean spectrum with standard deviation (ribbon) of infected samples centered and scaled on uninfected controls demonstrating discriminatory peaks at 710, 855, 1004, 1235, 1375 and 1505 cm^-1^.

The mean spectra acquired in infected and control (uninfected) urine samples are displayed in Fig. 2a. Spectral differences were highlighted by centering and scaling the spectra of infected samples referenced on uninoculated negative controls (as depicted in Fig. 2b). Biochemical assignment of peaks contributing to discrimination of infected from uninfected samples are presented in Table 2. PC-LDA classification performance is presented in Table 3. Furthermore, following application of PC-LDA, infected and uninfected samples were identified with 91.1% accuracy [95% CI 83.2–96.0%; *p* value < 0.05], corresponding to a sensitivity and specificity of 97% and 80% respectively (Table 3).

**Table 2:** Assignment of spectral peaks contributing to discrimination of infected samples from uninfected controls.

Raman peak (cm^−1^**)**	Assignment	References
710	Phospholipids and lipids	^33^
855	Phenylalanine, proline and tyrosine	^33,34^
	C–C stretching	^33^
1004	Phenylalanine and tyrosine	^18,34^
	CH_2_ scissoring	^34^
1235	Amide III	^18,33^
1375	Lipid CH_3_ deformation	^33^
1505	N = H bending	^33^

**Table 3:** Classification of infected and uninfected urine samples achieved using PC-LDA on the acquired Raman spectra.

Predicted	Actual	
	Control	Infected
**Control**	24	2
**Infected**	6	58

Accuracy 91.1% (CI: 83.2–96.0%; p-value < 0.05), Cohen’s K = 0.79. Sensitivity 97%, Specificity 80%.

#### Bacterial classification

**Figure 3:**
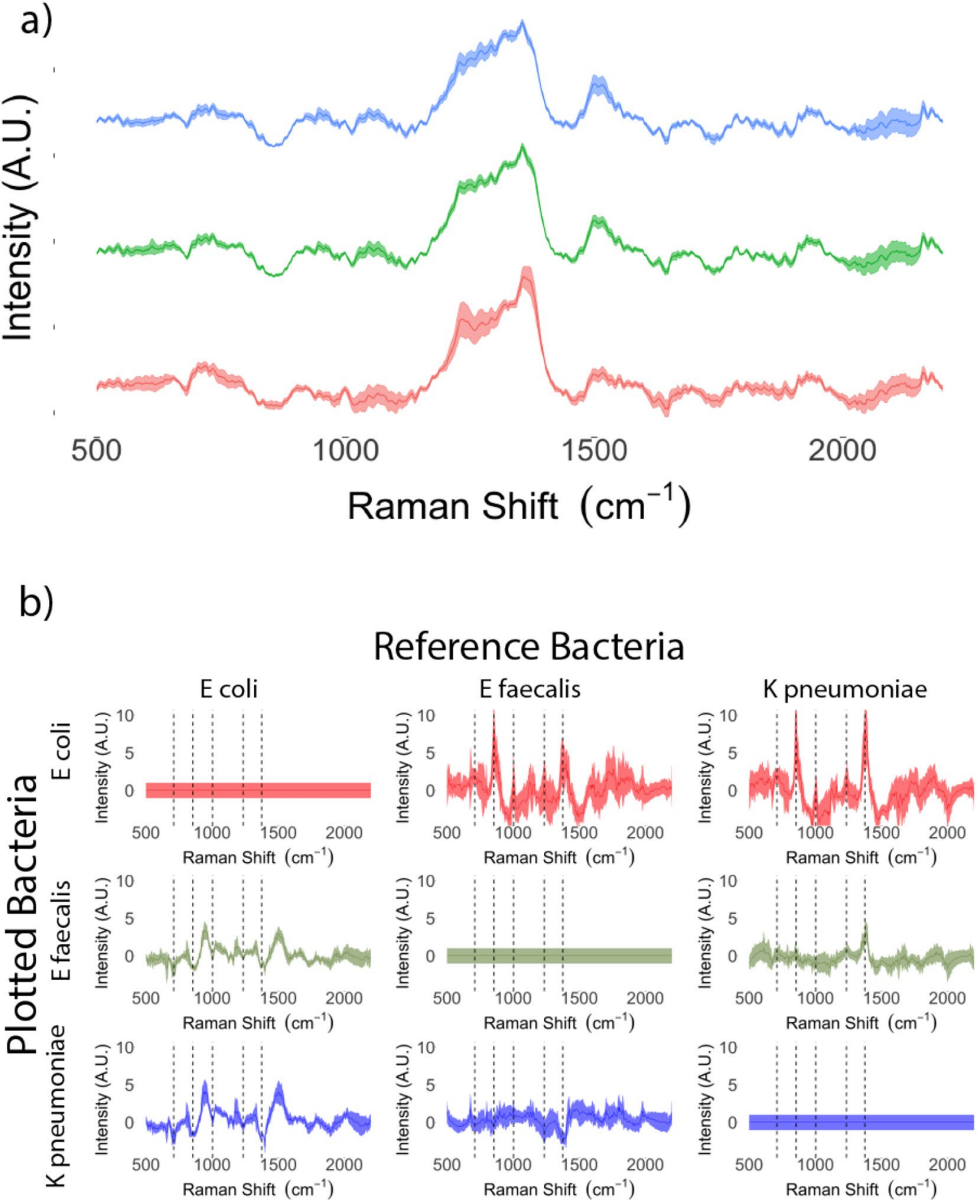
Identification of bacteria based on Raman spectra captured on SERS-active filters. (**a**) Mean Raman spectra with standard deviations (shaded areas) for *Escherichia coli* (red), *Enterococcus faecalis* (green) and *Klebsiella pneumoniae* (blue) captured on SERS-active filters. b) Spectra of individual species scaled and centered on one another demonstrating discriminatory peaks at 710, 855, 1004, 1235 and 1375 cm^-1^ (indicated by dotted lines).

**Figure 4:**
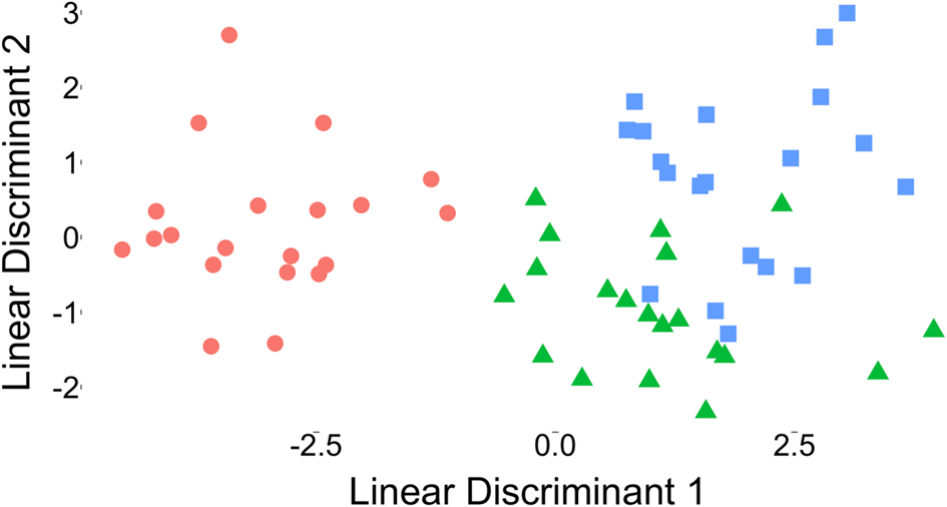
PC-LDA performed on Raman spectra from urine samples inoculated with *Escherichia coli* (red circle), *Enterococcus faecalis* (green triangle) and *Klebsiella pneumoniae* (blue square).

The mean spectra of the three uropathogen species are presented in Fig. 3a. Discriminating peaks at 710, 855, 1004, 1235 and 1375 cm^-1^ are demonstrated in plots of bacterial species scaled and centered on each other in Fig. 3b. The PC-LDA classification performance for bacterial identification is presented in Tables 4 and 5 and plotted graphically in Fig. 4. Amongst positive samples, the PC-LDA classification procedure provided clear discrimination of the three bacterial species (Fig. 4) and was able to identify the bacteria present in the samples with an accuracy of 91.6% (95% CI 81.6–97.2%; *p* value < 0.05; see Table 4). Furthermore, the sensitivity and specificity for identification of each bacterial species ranged from 86–100% (see Table 5).

**Table 4:** Bacterial classification in infected urine samples (achieved using PC-LDA). Accuracy 91.6% (95% CI: 81.6–97.2%; *p*-value < 0.05), Cohen's K = 0.88.

Predicted	Actual		
	*Escherichia coli*	*Enterococcus faecalis*	*Klebsiella pneumoniae*
***Escherichia coli***	20	0	0
***Enterococcus faecalis***	0	18	3
***Klebsiella pneumoniae***	0	2	17

**Table 5:** Classification statistics for PC-LDA-based identification of bacterial species in infected samples.

	*Escherichia coli*	*Enterococcus faecalis*	*Klebsiella pneumoniae*
Sensitivity	100%	90%	85%
Specificity	100%	93%	95%
Positive predictive value	100%	86%	90%
Negative predictive value	100%	95%	93%

### Discussion

This work demonstrates that SERS-active filters can provide rapid and accurate uropathogen classification in human urine samples containing clinically relevant concentrations of bacteria (10^5^ CFU/ml). This suggests the potential to alter the current diagnostic paradigm for UTIs by allowing for precision antimicrobial therapy from the point of diagnosis. The 91.1% detection accuracy of infected samples provided by SERS-active filters exceeds currently implemented UTI screening tests such as urinalysis^7,8,10,15^. Superior accuracy to current screening tests has the potential to reduce delayed diagnosis, while also avoiding the development of AMR through overtreatment.

The uropathogen classification accuracy of 91.6% approaches current gold standard classification with M,C&S and comparable to that of UTI diagnostic technologies under development including multiplex polymerase chain reaction and mass spectrometry^35–37^. Furthermore, sample processing was completed in minutes using widely available and low-cost equipment, indicating the potential for this approach to be developed into a point-of-care test in the future. Crucially, the ability to provide immediate bacterial classification would address a significant diagnostic need that is not addressed by existing technologies.

SERS has attracted much research attention as a result of its ability to provide accurate uropathogen classification without the need for prior amplification of the bacterial concentration through cell culture. SERS mediated uropathogen identification has been achieved with accuracies of up to 95.8% (e.g.^18,21,34,38–44^). However, circumventing bacterial amplification has required complex processes such as immunocapture, dielectrophoresis and use of optical tweezers to capture or aggregate pathogens, which are technically challenging or require expensive equipment^39,45^.

In this work, a uropathogen classification accuracy of 91.6% was achieved, while physical processing was limited to vacuum filtration therein completing physical processing and Raman spectral capture in less than 15 min per sample. Vacuum filtration is a widely used microbiology technique to concentrate pathogens from large sample volumes using widely available and low-cost equipment, with processing completed minutes^46^. Moreover, as vacuum filtration allows for rapid separation of uropathogens from urine, direct classification was achieved in urine samples rather than in suspensions or precultured samples. Additionally, a handheld Raman spectrometer was used in this study rather than the Raman microscopes commonly used in other SERS work^18,34,38–40,43^. While Raman microscopes provide high-resolution spectra, the high costs, large footprints and requirements for technical expertise preclude their incorporation into point-of-care devices. This work complements a growing SERS literature base that aims to provide rapid uropathogen classification without the need for complex physical processing or expensive microscopes^19,22,42^.

Many SERS mediated uropathogen classification studies have employed nanoparticle colloids to achieve surface enhancement^34,44^. Ensuring nanoparticle consistency is technically challenging, requiring stringent experimental control, while avoiding particle aggregation necessitates controlled storage and prompt use^47,48^. In this work, the SERS-active surface was applied through PVD—using a commercially available deposition system (Korvus Hex)—with close control of the metal thickness achieved using the deposition system’s control software. This fine control was used to assess multiple gold thicknesses, thereby allowing optimization of the surface enhancement effect. Lastly, in addition to the relative simplicity of the fabrication procedure, a further potential advantage of this approach is that the SERS-active filters are unlikely to require specialized storage or urgent use, and so may potentially be prepared in advance for use in point-of-care testing^49^.

This work demonstrates proof of principal that SERS-active filters can capture pathogens directly from urine and provide Raman signal enhancement therein allowing for identification of infected samples and pathogen classification, however limitations are present. Pre-filtered urine from healthy volunteers was used in phantoms samples so as to avoid sample contamination. While this provided a simplified background solution without human sediment in this research, a similar background solution can be achieved in clinical samples through dual filtration to initially remove human sediment with a larger pore filter to capture sediment prior to pathogen capture on SERS-active filters. Additionally, in this research positive samples were constrained to three reference strain bacteria at a set concentration so as to minimize intra-class variation. Clinical samples contain a larger number of bacterial species of varying strain and concentration providing a greater classification challenge. As such, application to clinical samples may yield lower diagnostic accuracy, although this may potentially be mitigated by a larger training set. Similarly, clinical samples frequently have multiple pathogens which will provide a significantly more challenging classification. While this is likely to reduce the classification accuracy, it is less likely to affect the identification of positive samples which is arguably more valuable clinical information.

### Conclusions

SERS-active filtration using gold coated membrane filters is a simple and rapid means of capturing bacteria direct from urine while providing significant Raman signal enhancement for pathogen identification. Applied to the diagnosis of UTIs in phantom urine samples, the technology provides accurate infection detection and bacterial classification with simple processing that can be completed in minutes. Thus, in the future, it may be possible to apply this approach to clinical samples to provide rapid, point-of-care bacterial identification, thereby permitting precision antimicrobial management.

## Methods

### Application of gold nanocoating

**Figure 5:**
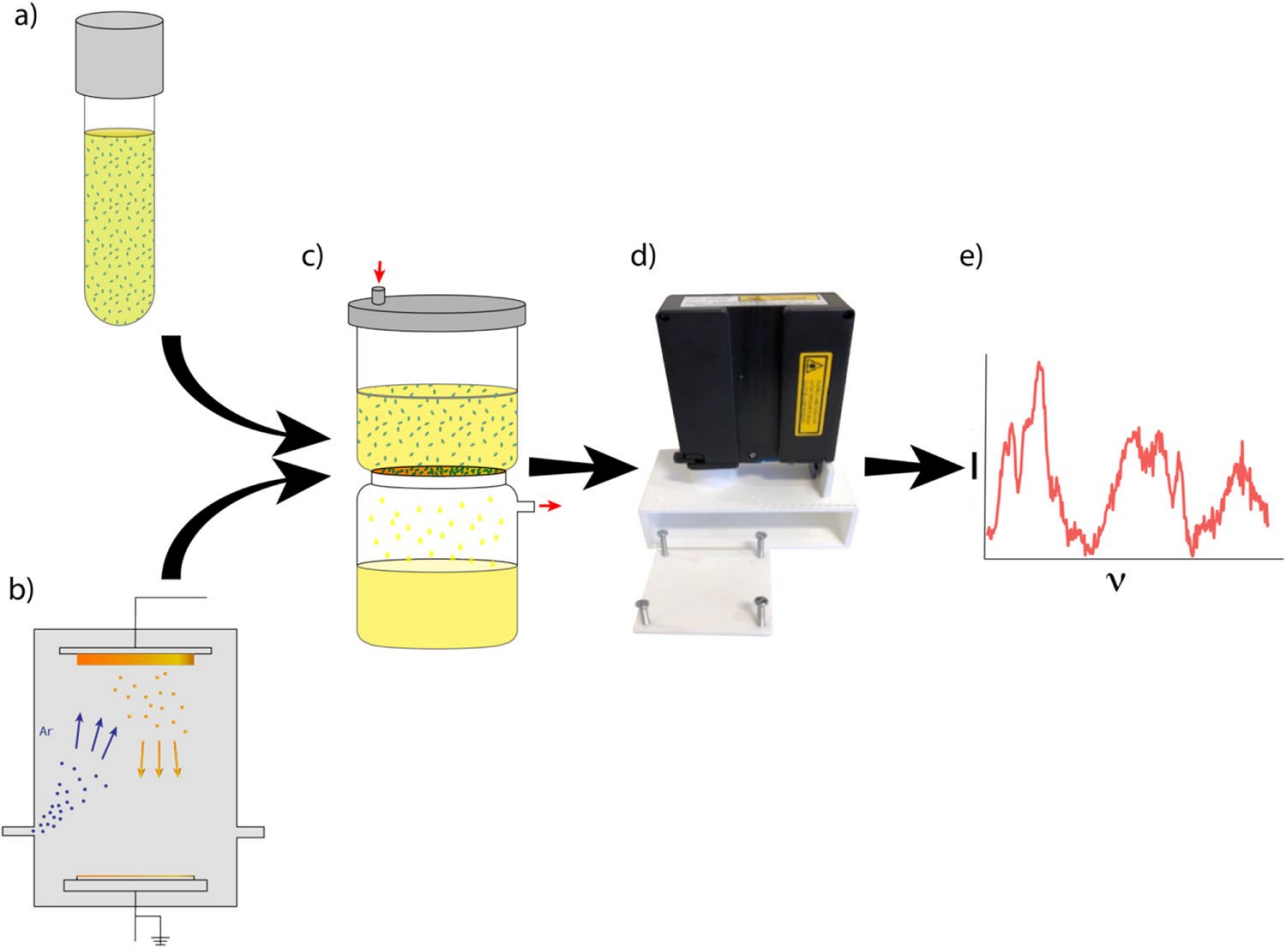
Methods overview. (**a**) Phantom urine samples were prepared by inoculating uropathogens (illustrated as blue ovals) into urine samples from healthy volunteers. (**b**) SERS-active filters were prepared by applying a 50 nm gold coating to polyvinylidene fluoride membrane filters using physical vapour deposition. (**c**) Vacuum filtration captured the uropathogens from urine and applied them directly to the SERS-enhancing surface (Red arrows indicate direction of vacuum). (**d**) Raman spectra were collected with a handheld spectrometer supported by a custom 3D-printed holder. (**e**) The spectra were plotted and analyzed (I—intensity, ν—wavenumber).

SERS-active membrane filters were created by applying a gold coating to commercially available membrane filters (0.45 μm pore size) using PVD. PVDF membrane filters were selected for this purpose as they have previously been demonstrated to have a simple baseline Raman spectrum with minimal variability, and therefore contribute minimal background signal^50^. In order to establish the gold thickness for optimal Raman signal enhancement, different layer thicknesses were fabricated and assessed. Gold coatings were applied to the membrane filters (see Fig. 5b and Supplemental Fig. 1) using a Korvus Hex Deposition System using DC magnetron sputtering with a 2″ gold target onto a 4″ rotating stage. In addition to unsputtered controls, coating thicknesses of 2.5, 5, 7.5, 10, 12.5, 15, 20, 25, 50, 100 and 150 nm were applied. Coating thicknesses were controlled using quartz crystal monitoring.

#### SERS enhancement optimization and assessment

The Raman signal enhancement gained by differing gold coating thicknesses was assessed using Rhodamine 6G. A Rhodamine 6G solution was prepared and diluted in deionized water to a final concentration of 1 µM. 100 µl of Rhodamine 6G was then pipetted onto each membrane filter. Raman spectra were collected from 10 separate filters for each coating thickness using an IDRaman mini 2.0 (Ocean Insight) with a point-and-shoot attachment. A custom, 3D-printed mount was used to position the spectrometer at the optimal focal distance (as described in previous work^50^; also see Supplemental Fig. 2). For each filter, 5 spectra were collected (over the range 400–2300 cm^−1^) and averaged with 5 s acquisition times using a laser excitation power of 10 mW at 785 nm. The spectra were preprocessed by Savitsky-Golay filtering and background subtraction of a 7^th^ order polynomial^51^ (see Supplemental Fig. 3).

### Diagnostic phantom UTIs

#### Phantom UTI samples

The diagnostic performance of the SERS-active membrane filters was assessed using phantom urine infection samples (Fig. 5a). Reference strain *Escherichia coli* ATCC25922, *Enterococcus faecalis* ATCC29212 and *Klebsiella pneumoniae* ATCC31883 were acquired from North West London Pathology Service and cultured on Columbia blood agar at 37º Celsius for 24 h. Harvested colonies were stored on a Microbank cryopreservation system at − 70º Celsius until required.

To create a phantom UTI sample a single Microbank bead was cultured in 3 ml brain–heart infusion at 37º Celsius for 24 h. The cultured bacteria were centrifuged at 11,000 rpm for 1 min. The resulting supernatant was discarded, and the bacterial pellet resuspended in phosphate buffered solution (PBS). This centrifuge and resuspension process was repeated 5 times so as to remove remaining culture medium^52^. The absorption at 600 nm was used to dilute the resulting bacterial suspension down to a 0.5 McFarland Standard in PBS, corresponding to a bacterial load of 1.5 × 10^8^ CFU/ml. The resulting bacterial suspension was serially diluted to 10^5^ CFU/ml in urine acquired from healthy human volunteers, which had been pre-filtered through a 0.2 μm pore size Corning vacuum filtration system to remove any human cellular material or contaminating microbes. This process culminated in cultured and washed uropathogens diluted to a clinically relevant concentration of 10^5^ CFU/ml in human urine. A total of 90 samples were analyzed including 20 of each uropathogen and 30 controls (uninoculated healthy urine). Urine was collected from healthy volunteers with their informed consent, and in accordance with Health Research Authority and Research Ethics Committee approval (IRAS: 237,195, REC: 18/LO/0314. Bromley Research Ethics committee). This work followed the principles of the World Medical Association’s Declaration of Helsinki. All microbiologic work was undertaken in a containment level 2 laboratory.

#### Sample processing and Raman capture

30 ml Phantom urine samples underwent vacuum filtration through the SERS-active filters (50 nm gold coating, which provided the optimal signal enhancement. See Fig. 5c). An insert was designed and 3D-printed for vacuum filtration system which reduced the filter area from a diameter of 47 mm down to 10 mm (Supplemental Fig. 4). These inserts provided a number of benefits. Firstly, while the stage of the Korvus Hex will only allow for sputtering of four 47 mm filters per cycle, it can fit a single 90 mm filter which can subsequently be divided to provide over forty 10 mm filters. This is important as each coating cycle takes over an hour. Secondly, the uropathogens were concentrated into a smaller area, leading to a 22 times greater concentration. Finally, the insert allows for an additional upper layer housing a wider pore-size filter (Supplemental Fig. 4). This dual filtration will be integral in future work with clinical samples as large pore-size filters will remove patient cells and debris while allowing pathogens through to be captured on the SERS-active filters. Excess urine was washed off by subsequently vacuum filtering 10 ml PBS through the SERS-active filters. Thereafter, the SERS-active filters were removed from the vacuum filtration system and air dried prior to Raman capture. Physical processing was completed in under 10 min.

The processed SERS-active filters were transferred to a 3D-printed mounting system (described in previous work^50^, and demonstrated in Supplemental Fig. 2), which acted to secure the samples at the focal point of the Raman spectrometer (Fig. 5d). Raman spectra were acquired (Fig. 1e) using an IDRaman mini 2.0 (Ocean Insight) handheld spectrometer (excitation wavelength—785 nm; laser power—50 mW). Raman spectra were collected across a range of 400–2300 cm^-1^ with a spectral resolution of 12–14 cm^-1^(technical specifications of the IDRaman mini 2.0 are available in Supplemental Table 1). For each filter (sample), 60 spectra were collected and averaged with a 3 s acquisition time per spectrum (providing a total acquisition time of 3 min for each sample). The reference spectrum and raster orbital scanner functions of the spectrometer were used in all cases.

### Spectral processing and analysis

The Raman spectra acquired from the filters were processed with R scripts (version 3.5.1) developed in-house (Fig. 5e). The spectra were first truncated from 500 to 2200 cm^-1^ (as this range contained all visually discernible spectral peaks). High frequency noise and cosmic spikes were removed with a Savitzky-Golay filter before background subtraction using cubic spline interpolation. The background corrected spectra were then vector normalized by dividing each spectrum by its hypergeometric Euclidean distance (*d*), the square root of the sum of the squared vector components (i.e. the intensity values at each wavelength, *p*_*i*_) (Eq. 1). Spectral preprocessing is demonstrated in Supplemental Fig. 3.

1 $$d\left( p \right) = \sqrt {\mathop \sum \limits_{i}^{n} p_{i}^{2} }$$

The mean Raman spectra were plotted for infected and control samples, as well as for individual bacterial species. Sample classification was performed using Principal Component—Linear Discriminant Analysis (PC-LDA). Dimensionality reduction was achieved using principal component analysis and feature extraction, and the reduced features were then subjected to classification using linear discriminant analysis. To assess the diagnostic potential, the classification results were used to calculate the sensitivity, specificity and overall diagnostic accuracy for identification of the individual bacterial species and for identification of infected vs. uninfected samples. Finally, 95% confidence intervals (CIs), *p* values and Cohen’s K values were calculated to provide assessments of the statistical significance of the classification and the degree of agreement with the ground truth (i.e. the actual infection statuses or the actual bacterial species).

## Supplementary Information

Supplementary Information

## Data Availability

The data and code are made available to the editorial board and referees, including SERS coating Thickness dataset, the phantoms urine dataset and the R script.

## Abbreviations

AMR: Antimicrobial resistance
M,C&S: Microscopy culture and sensitivity
PBS: Phosphate buffered solution
PCA: Principal component analysis
PC-LDA: Principal component–linear discriminant analysis
PVD: Physical vapour deposition
PVDF: Polyvinylidene Fluoride
SERS: Surface enhanced Raman spectroscopy

## References

[1] Ozturk R, Murt A (2020). Epidemiology of urological infections: A global burden. World J. Urol..

[2] Stamm WE, Norrby SR (2001). Urinary tract infections: Disease panorama and challenges. J. Infect. Dis..

[3] Flores-Mireles AL, Walker JN, Caparon M, Hultgren SJ (2015). Urinary tract infections: Epidemiology, mechanisms of infection and treatment options. Nat. Rev. Microbiol..

[4] Foxman B (2014). Urinary tract infection syndromes. Infect. Dis. Clin. North Am..

[5] Scholes D (2005). Risk factors associated with acute pyelonephritis in healthy women. Ann. Intern. Med..

[6] Mayr FB, Yende S, Angus DC (2014). Epidemiology of severe sepsis. Virulence.

[7] Schmiemann G, Kniehl E, Gebhardt K, Matejczyk MM, Hummers-Pradier E (2010). The diagnosis of urinary tract infection: A systematic review. Dtsch. Arztebl. Int..

[8] Van Nostrand JD, Junkins AD, Bartholdi RK (2000). Poor predictive ability of urinalysis and microscopic examination to detect urinary tract infection. Am. J. Clin. Pathol..

[9] Little, P. *et al.* Dipsticks and diagnostic algorithms in urinary tract infection: Development and validation, randomised trial, economic analysis, observational cohort and qualitative study. *Health Technol. Assess* **13**, 3–4, 9-11, 1–73. 10.3310/hta13190 (2009). 10.3310/hta13190 19364448

[10] D’eVille WL (2004). The urine dipstick test useful to rule out infections. A meta-analysis of the accuracy. BMC Urol..

[11] Broeren MA, Bahceci S, Vader HL, Arents NL (2011). Screening for urinary tract infection with the Sysmex UF-1000i urine flow cytometer. J. Clin. Microbiol..

[12] Llor C, Bjerrum L (2014). Antimicrobial resistance: Risks associated with antibiotic overuse and initatives to reduce the problem. Ther. Adv. Drug. Saf..

[13] Melander RJ, Zurawski DV, Melander C (2018). Narrow-spectrum antibacterial agents. Medchemcomm.

[14] Messacar K (2018). Narrow-spectrum, compared with broad-spectrum, antibiotics equally effective with less adverse events. J. Pediatr..

[15] Davenport M (2017). New and developing tehnologies for urinary tract infections. Nat. Urol..

[16] Jarvis RM, Goodacre R (2008). Characterisation and identification of bacteria using SERS. Chem. Soc. Rev..

[17] Kastanos E, Kyriakides A, Hadjigeorgiou K, Pitris C (2012). A novel method for bacterial UTI diagnosis using Raman spectroscopy. Int. J. Spectrosc..

[18] Kloss S (2013). Culture independent Raman spectroscopic identification of urinary tract infection pathogens: A proof of principle study. Anal. Chem..

[19] Oliviera F, Giana H, Silviera L (2012). Discrimination of selected species of pathogeni bacteria using near-infrared Raman spectroscopy and prinicipal component analysis. J. Biomed. Opt..

[20] Pahlow S (2015). Isolation and identification of bacteria by means of Raman spectroscopy. Adv. Drug. Deliv. Rev..

[21] Tien N (2018). Diagnosis of bacterial pathogens in the urine of urinary-tract-infection patients using surface-enhanced Raman spectroscopy. Molecules.

[22] Yang D, Zhou H, Dina NE, Haisch C (2018). Portable bacteria-capturing chip for direct surface-enhanced Raman scattering identification of urinary tract infection pathogens. R. Soc. Open Sci..

[23] Liu TY (2011). Functionalized arrays of Raman-enhancing nanoparticles for capture and culture-free analysis of bacteria in human blood. Nat. Commun..

[24] Mircescu NE (2014). Towards a receptor-free immobilization and SERS detection of urinary tract infections causative pathogens. Anal. Bioanal. Chem..

[25] Mosier-Boss PA (2017). Review on SERS of bacteria. Biosensors (Basel).

[26] Premasiri WR (2005). Characterization of surface enhanced Raman scattering of bacteria. J. Phys. Chem..

[27] Lin C-C (2014). A filter-like AuNPs@MS SERS substrate for *Staphylococcus aureus* detection. Biosens. Bioelectron..

[28] Lee CH, Hankus ME, Tian L, Pellegrino PM, Singamaneni S (2011). Highly sensitive surface enhanced Raman scattering substrates based on filter paper loaded with plasmonic nanostructures. Anal. Chem..

[29] Fateixa S, Raposo M, Nogueira HIS, Trindade T (2018). A general strategy to prepare SERS active filter membranes for extraction and detection of pesticides in water. Talanta.

[30] Rule Wigginton K, Vikesland PJ (2010). Gold-coated polycarbonate membrane filter for pathogen concentration and SERS-based detection. Analyst.

[31] Szymborski T, Witkowska E, Adamkiewicz W, Waluk J, Kaminska A (2014). Electrospun polymer mat as a SERS platform for the immobilization and detection of bacteria from fluids. Analyst.

[32] Jensen L, Schatz GC (2006). Resonance Raman scattering of rhodamine 6g as calculated using time-dependent density functional theory. J. Physc. Chem. Lett..

[33] Movasaghi Z, Rehman S, Rehman IU (2007). Raman spectroscopy of biological tissues. Appl. Spectrosc. Rev..

[34] Avci E, Kaya NS, Ucankus G, Culha M (2015). Discrimination of urinary tract infection pathogens by means of their growth profiles using surface enhanced Raman scattering. Anal Bioanal Chem.

[35] Ferreira L, Sanchez-Juanes F, Munoz-Bellido JL, Gonzalez-Buitrago JM (2011). Rapid method for direct identification of bacteria in urine and blood culture samples by matrix-assisted laser desorption ionization time-of-flight mass spectrometry: Intact cell vs. extraction method. Clin. Microbiol. Infect..

[36] Veron L (2015). Rapid urine preparation prior to identification of uropathogens by MALDI-TOF MS. Eur. J. Clin. Microbiol. Infect. Dis..

[37] Lehmann LE (2011). Rapid qualitative urinary tract infection pathogen identification by SeptiFast real-time PCR. PLoS ONE.

[38] Premasiri WR (2017). Rapid urinary tract infection diagnostics by surface-enhanced Raman spectroscopy (SERS): Identification and antibiotic susceptibilities. Anal. Bioanal. Chem..

[39] Schroder UC (2013). Combined dielectrophoresis-Raman setup for the classification of pathogens recovered from the urinary tract. Anal. Chem..

[40] Schroder UC (2015). Rapid, culture-independent, optical diagnostics of centrifugally captured bacteria from urine samples. Biomicrofluidics.

[41] Jarvis RM, Goodacre R (2004). Ultra-violet Raman spectroscopy for rapid discrimination of urinary tract infection bacteria. FEMS Microbiol. Lett..

[42] Kastanos EK, Kyriakides A, Hadjigeorgiou K, Pitris C (2010). A novel method for urinary tract infection diagnosis and antibiogram using Raman spectroscopy. J. Raman Spectrosc..

[43] Goodacre R (1998). Rapid identification of urinary tract infection bacteria using hyperspectral whole-organism fingerprinting and artificial neural networks. Micrbiology.

[44] Nordstrom, R. J. *et al.* In *Optical Diagnostics and Sensing XII: Toward Point-of-Care Diagnostics; and Design and Performance Validation of Phantoms Used in Conjunction with Optical Measurement of Tissue IV* (2012).

[45] Xie C (2005). Identification of single bacterial cells in aqueous solution using confocal laser tweezers Raman spectroscopy. Anal. Chem..

[46] Harrigan WF, McCance ME (1966). Laboratory methods in microbiology.

[47] Haynes CL, McFarland AD, Van Duyne RP (2005). Surface enhanced Raman spectroscopy. Anal. Chem..

[48] Lane LA, Qian X, Nie S (2015). SERS nanoparticles in medicine: From label-free detection to spectroscopic tagging. Chem. Rev..

[49] Mattox, D. M. *Handbook of Physical Vapour Deposition (PVD) Processing*. 944 (1998).

[50] Dryden, S. *et al.* in *Optical Diagnostics and Sensing XX: Toward Point-of-Care Diagnostics* (2020).

[51] Ma J, Zhang Q, Lin K, Zhou L, Ni Z (2018). Piezoelectric and optoelectronic properties of electrospinning hybrid PVDF and ZnO nanofibers. Mater. Res. Express.

[52] Premasiri WR, Gebregziabher Y, Ziegler LD (2011). On the difference between surface-enhanced Raman scattering (SERS) spectra of cell growth media and whole bacterial cells. Appl. Spectrosc..

