# Supplementary material for: Rapid uropathogen identification using surface enhanced Raman spectroscopy active filters
Source: Sci Rep. 2021 Apr 22;11:8802. doi: 10.1038/s41598-021-88026-9 (PMC8062667; doi:10.1038/s41598-021-88026-9)
Supplement: Supplementary file 1 — Supplementary Information [file 41598_2021_88026_MOESM1_ESM.pdf]

SUPPLEMENTARY INFORMATION

Rapid Uropathogen Identification Using Surface Enhanced Raman Spectroscopy Active Filters

Simon D. Dryden[1*], Salzitsa Anastasova[2], Giovanni Satta[3], Alex J. Thompson[1,2*ǂ], Daniel R. Leff[1,2ǂ], and Ara Darzi[1,2ǂ]

[1]Department of Surgery and Cancer, Imperial College London, London, W2 1NY, United Kingdom
[2]Hamlyn Centre for Robotic Surgery, Imperial College London, London, SW1 2AZ, United Kingdom
[3]Department of Infection, Imperial College NHS Trust, London, W6 8RF, United Kingdom

[*]**Correspondence:**
Simon D. Dryden, 10th Floor, QEQM Wing, Department of Surgery and Cancer, Imperial College London, St Mary's Hospital, W2 1NY, London, United Kingdom
Simon.dryden@imperial.ac.uk
Alex J. Thompson, 2nd Floor, Paterson Building, Department of Surgery and Cancer, Imperial College London, St Mary's Hospital, W2 1NY, London, United Kingdom
Alex.thompson08@imperial.ac.uk

[ǂ]these authors contributed equally to this work

Section 1: Photographs and scanning electron micrographs of coated filters

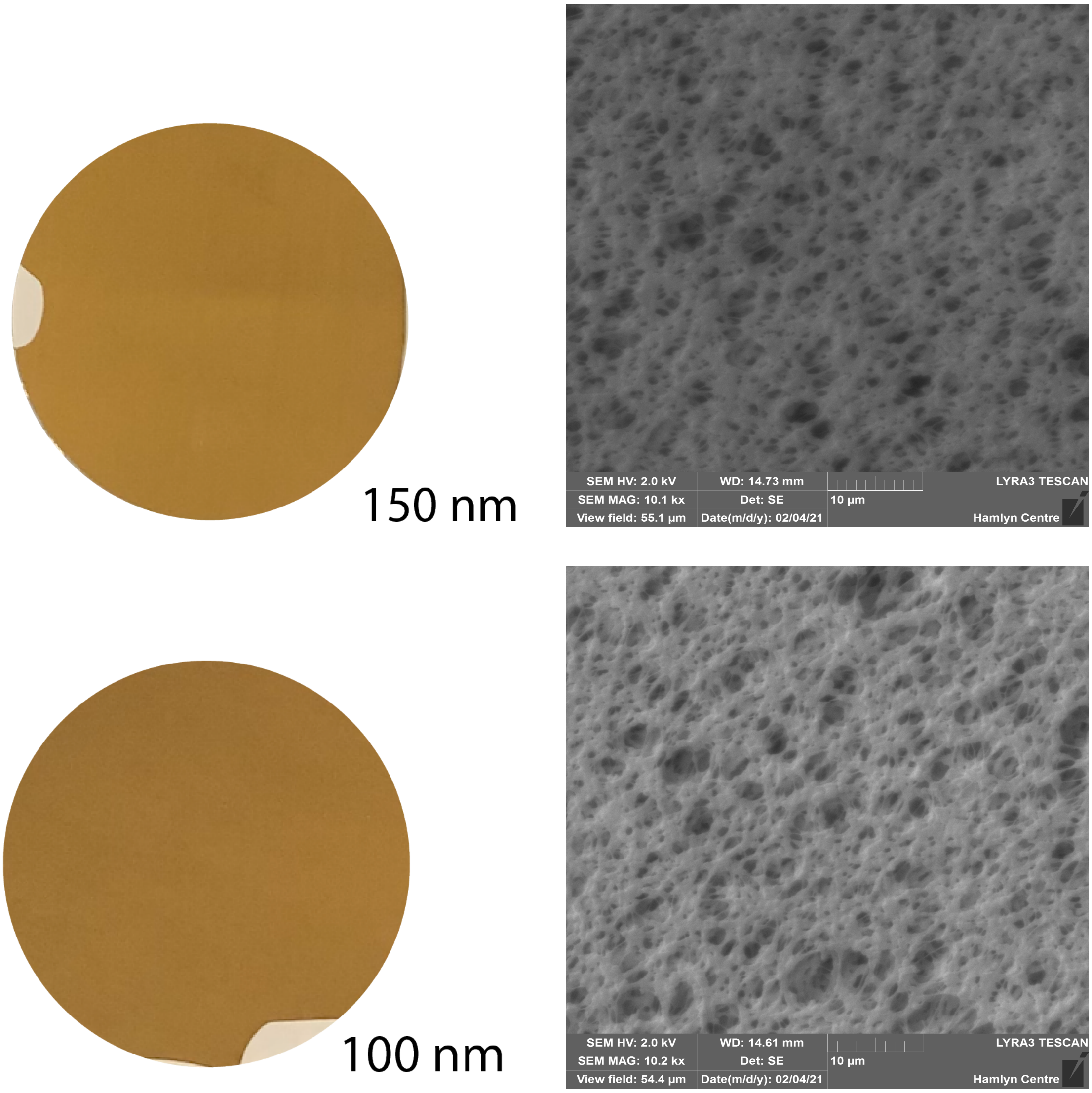

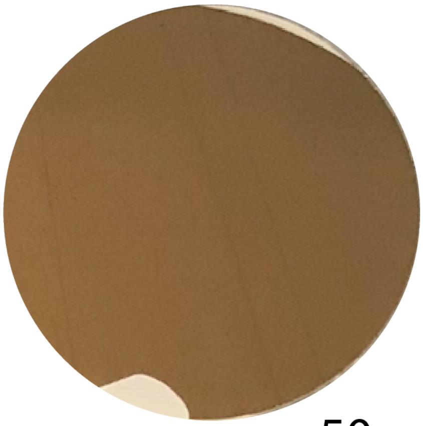

50 nm

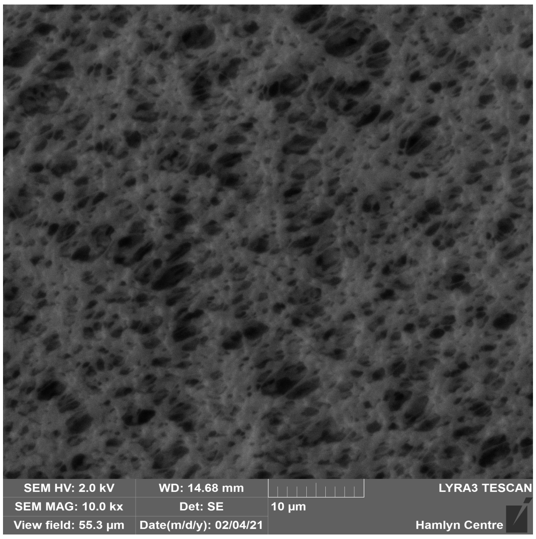

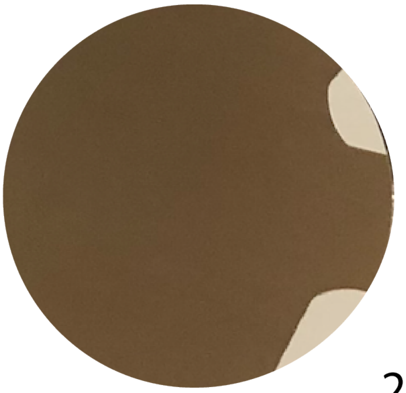

25 nm

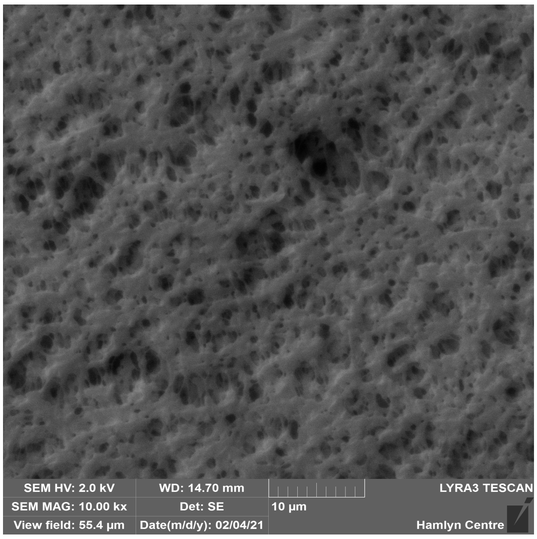

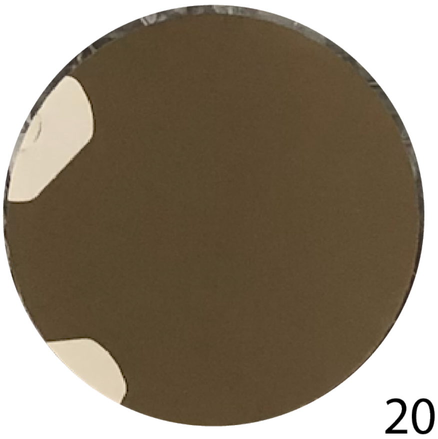

20 nm

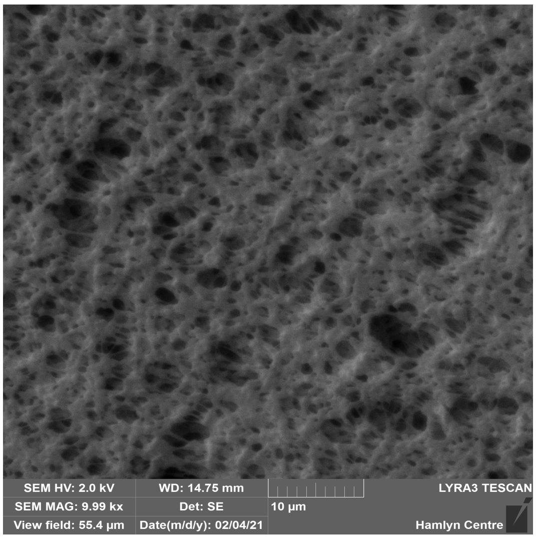

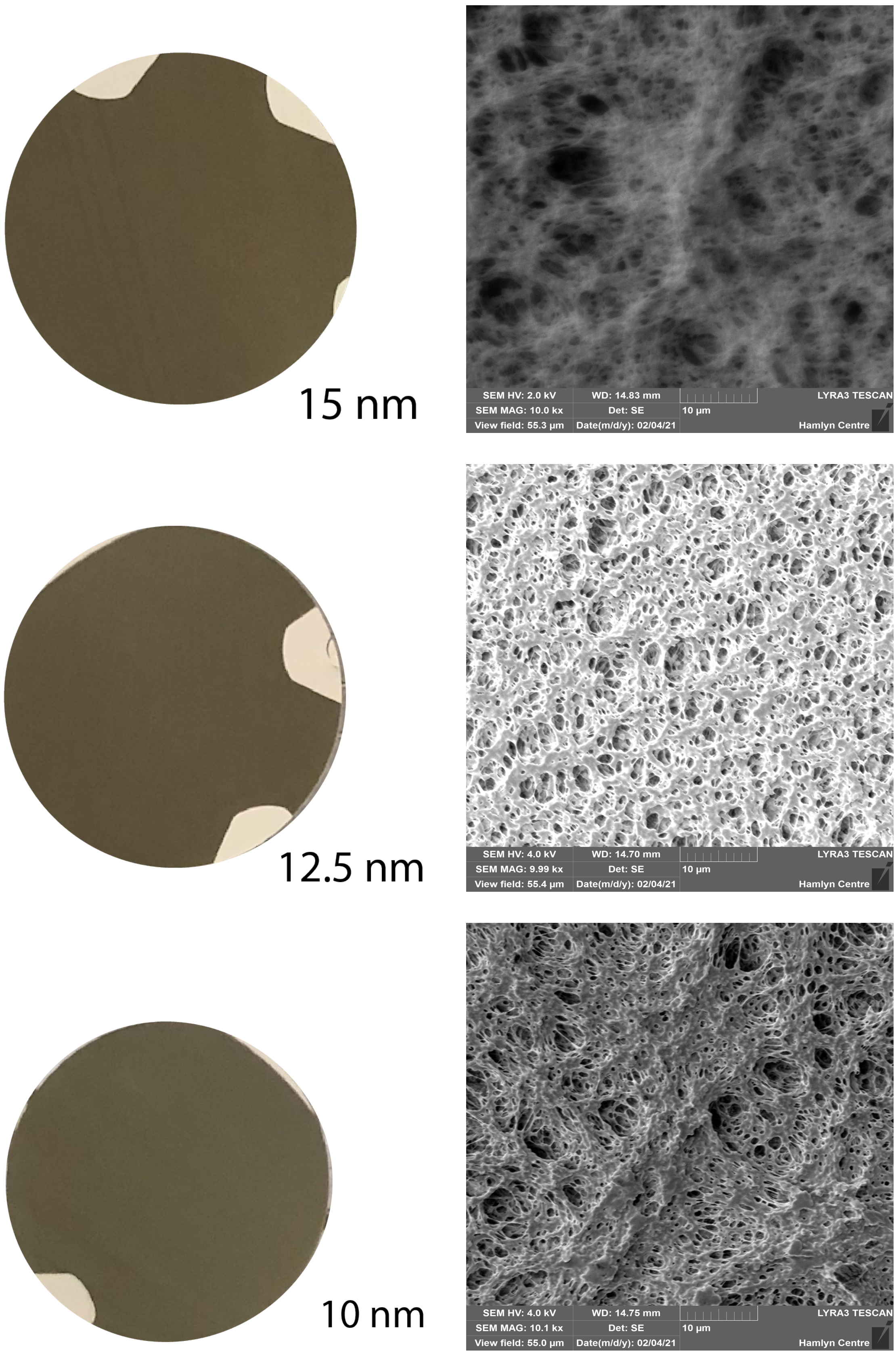
15 nm
SEM HV: 2.0 kV
WD: 14.83 mm
LYRA3 TESCAN
SEM MAG: 10.0 kx
Det: SE
10 μm
View field: 55.3 μm
Date(m/d/y): 02/04/21
Hamlyn Centre
12.5 nm
SEM HV: 4.0 kV
WD: 14.70 mm
LYRA3 TESCAN
SEM MAG: 9.99 kx
Det: SE
10 μm
View field: 55.4 μm
Date(m/d/y): 02/04/21
Hamlyn Centre
10 nm
SEM HV: 4.0 kV
WD: 14.75 mm
LYRA3 TESCAN
SEM MAG: 10.1 kx
Det: SE
10 μm
View field: 55.0 μm
Date(m/d/y): 02/04/21
Hamlyn Centre

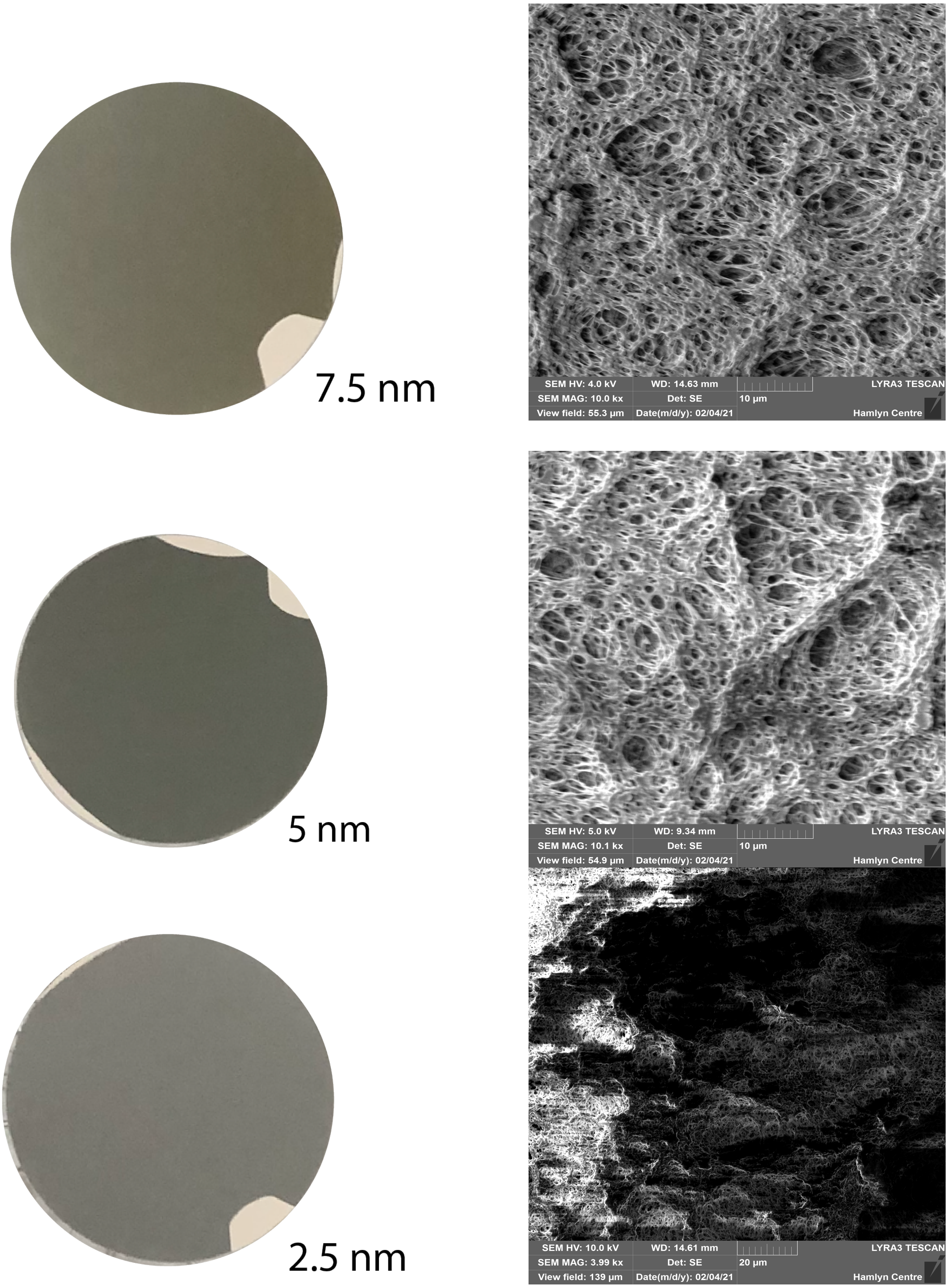

SUPPLEMENTAL FIGURE 1. Photographs and scanning electron micrographs of polyvinylidene fluoride membrane filters with gold coatings ranging from 2.5 nm to 150 nm.

Section 2: Spectrometer mount

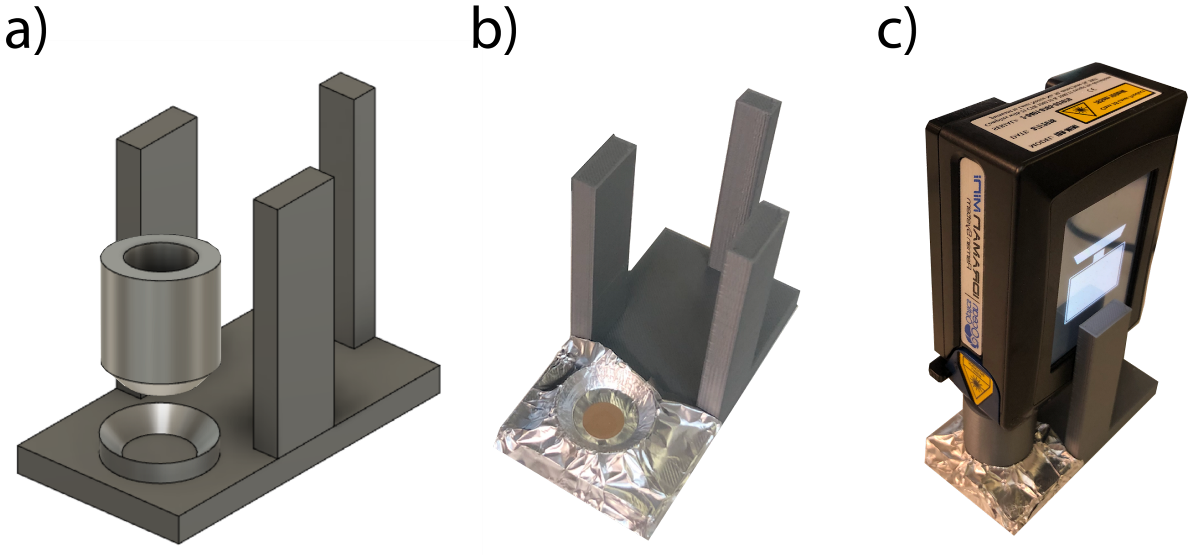

SUPPLEMENTAL FIGURE 2. A custom designed spectrometer mount provided a consistent focal length between the spectrometer and filters. a) Computer-aided design (CAD) model of spectrometer mount. b) 3D printed mount loaded with a gold coated filter. c) Ocean Optics IDRaman mini 2.0 in 3D printed mount.

Section 3: Spectral preprocessing

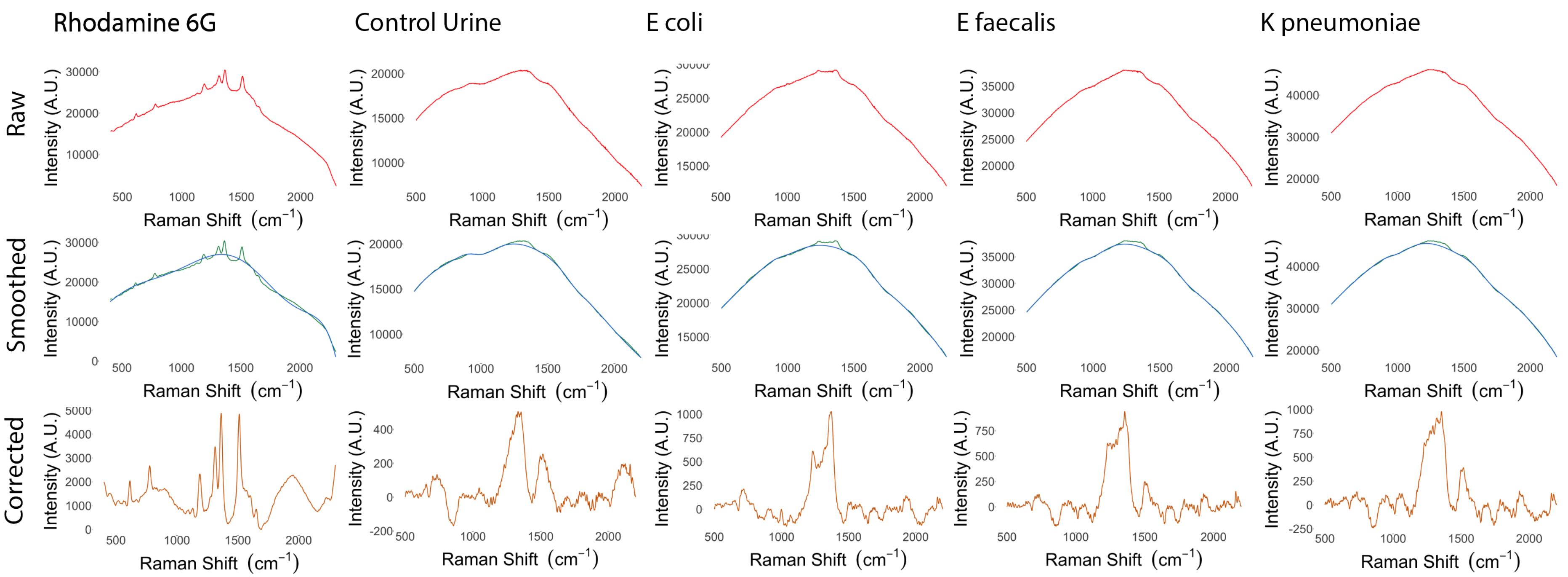

SUPPLEMENTAL FIGURE 3. Spectral preprocessing of typical spectra acquired from SERS-active filters with Rhodamine 6G, uninfected control urine, and urine with *E. coli*, *E. faecalis* and *K. pneumoniae*. The top row demonstrates the raw spectra of each. The second row demonstrates the spectra smoothed by passing a Savitsky-Golay smoothing filter prior to fitting a baseline. The third row demonstrates the spectra corrected by subtraction of the fitted baseline. Polynomial fitting (7th order) was used for background subtraction of Rhodamine 6G spectra. Cubic spline interpolation was used for background subtraction of urine and bacteria spectra. The latter provides a more accurate background subtraction but requires *a priori* manual identification of interpolant points from pilot spectra. The same interpolant points were used for all spectra. Thus, polynomial fitting was used for Rhodamine 6G spectra (for which spectral peaks were easily identifiable) while cubic spline interpolation was used for bacteria/urine spectra (which exhibited lower signal-to-noise ratios).

Section 4: Dual filtration system

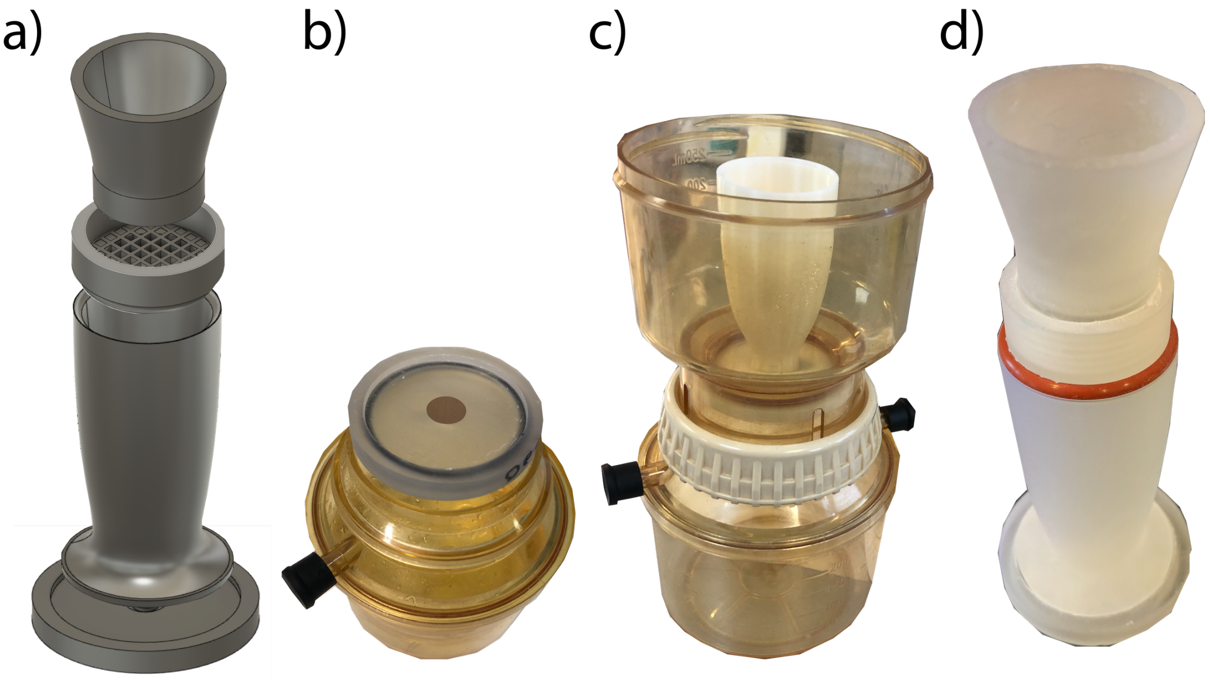

SUPPLEMENTAL FIGURE 4. A custom vacuum filtration insert was designed and 3D printed. The insert allowed for 10 mm filters to be inserted into a commercially available Sterifil vacuum filtration system. This system provided multiple advantages over the commercially available vacuum filtration system: multiple 10 mm filters could be gold-coated simultaneously; pathogens were concentrated into a smaller surface area; and an additional upper level loaded with a larger 3 μm pore size filter will allow for dual filtration in future work, therein providing a rapid single step method for separate removal of human cells while allowing capture of pathogens onto the SERS-active membranes. a) CAD model of the complete dual filtration system. b) A SERS-active filter loaded onto the 3D printed custom insert. c) The insert loaded into a commercially available vacuum filtration system. d) 3D printed insert with additional upper level allowing for dual filtration.

Section 5: Ocean Optics IDRaman mini 2.0 technical specifications

**Specifications**	**Criteria**
Raman Spectrum Range	400 to 2300 cm^{-1}
Spectral Resolution	12 to 14 cm^{-1} (FWHM) across spectral range
Laser (excitation wavelength)	785nm±0.5 nm, 2 cm^{-1} line width, stability <0.1 cm^{-1}
Laser Output Power	100 mW, 70 mW at the sample
Collection Optics	NA = 0.50, 8mm working distance; 0.2 to 2.5 mm spot size
Signal to Noise Ratio (SNR)	SNR = 1000; 70 mW toluene 0.1 second integration

SUPPLEMENTAL Table 1. Technical specifications of the Ocean Optics IDRaman mini 2.0 handheld spectrometer
